# Giant cell carcinoma of the urinary bladder: Clinicopathologic analysis and oncological outcomes

**DOI:** 10.1007/s00428-024-03858-w

**Published:** 2024-07-18

**Authors:** Frederico Portugal-Gaspar, Antonio Lopez-Beltran, Gladell P. Paner, Ana Blanca, Enrique Gómez Gómez, Rodolfo Montironi, Alessia Cimadamore, Andreia Bilé, Metka Volavšek, Liang Cheng

**Affiliations:** 1 Urology Department, Egas Moniz Hospital, Centro Hospitalar de Lisboa Ocidental, Lisbon, Portugal; 2 Department of Morphological Sciences, Cordoba University Medical School, E-14004, Cordoba, Spain; 3 Departments of Pathology and Surgery (Urology), University of Chicago, Chicago, IL USA; 4 Maimonides Biomedical Research Institute of Cordoba, E-14004, Cordoba, Spain; 5 Urology Department, Reina Sofía University Hospital, Maimonides Institute of Biomedical Research of Cordoba (IMIBIC), University of Cordoba (UCO), Cordoba, Spain; 6 Molecular Medicine and Cell Therapy Foundation, Department of Clinical and Molecular Sciences, Polytechnic University of the Marche Region, Ancona, Italy; 7 Institute of Pathological Anatomy, Department of Medicine (DMED), Udine University, 33100 Udine, Italy; 8 Institute of Pathology, Faculty of Medicine, University of Ljubljana, Ljubljana, Slovenia; 9 Department of Pathology and Laboratory Medicine, Department of Surgery/Urology, Warren Alpert Medical School of Brown University, Lifespan Health, and The Legorreta Cancer Center at Brown University, Providence, RI USA

**Keywords:** Bladder cancer, Progression, Giant cell carcinoma, Variant, Subtype

## Abstract

We present the clinicopathological features of 23 cases of the giant cell subtype of urothelial carcinoma, a rare subtype of bladder cancer recognized in the current World Health Organization classification of urological tumors. Histologically, the architectural pattern of the tumor varied from infiltrating to the solid expansile pleomorphic tumor with giant, bizarre, anaplastic cells. Typical or atypical mitotic figures were frequently present in all cases. Between 10 and 30% of the tumor had a giant cell component. All cases were associated with conventional high-grade urothelial carcinoma, with areas of squamous cell divergent differentiation and micropapillary carcinoma present in six and two cases, respectively. In one case each had sarcomatoid, nested, small cell, or glandular divergent differentiation. At diagnosis, 35% of patients had advanced disease and 12% had distant metastases. When comparing giant cell urothelial carcinoma with conventional urothelial carcinoma in a matched analysis, differences in overall and cancer-specific survival were observed, particularly in the T1 stage category. Immunohistochemical staining showed a similar profile of urothelial lineage with frequent positive expression of uroplakin II, GATA3, CK20, CK7, and S100P in both giant cell and conventional urothelial carcinomas. High Ki67 proliferation (range, 60–90%; mean, 71%) and nuclear p53 accumulation (mutant profile; range, 50–90%; mean, 64%) were observed. Using the 22C3 assay, the expression of PD-L1 was found to be variable in two cases, and beta-HCG was negative. In conclusion, giant cell carcinoma is a subtype of urothelial carcinoma associated with advanced clinical stage and a trend to lower survival rates.

## Introduction

Giant cell urothelial carcinoma (GCUC) is a rare and aggressive variant of urothelial carcinoma (UC) characterized by the presence of highly pleomorphic and bizarre tumor giant cells, similar to those seen in giant cell carcinoma of the lung [1–4]. This variant has been recognized in the current classification of urothelial neoplasms by the World Health Organization (WHO) [1]. However, the available data on the pathological and immunohistochemical characteristics and clinical behavior of GCUC are limited and are mainly derived from case reports or small case series [2, 4–8]. Giant cell carcinoma has been identified in a variety of organs, including the lung, upper urinary tract, ovary, pancreas, breast, kidney, liver, gallbladder, and prostate, with a similar degree of aggressiveness [9–18].

The characteristic features of GCUC include aggregates or sheets of mononucleated and multinucleated, highly pleomorphic, giant, bizarre cells [1, 2, 19]. These proliferating cells may appear undifferentiated [2, 20]. They are associated with variable tumor cell necrosis and cellular cohesion. Notably, the diagnosis excludes a spindle cell component [21, 22]. This is to avoid confusion with the sarcomatoid subtype of UC. The frequent expression of urothelial lineage markers, such as GATA3, on immunohistochemistry supports the urothelial origin [4, 23–29].

The differentiation of primary GCUC from poorly differentiated carcinomas, such as osteoclast-rich undifferentiated carcinoma, UC with trophoblastic giant cells, or large cell undifferentiated carcinoma, is crucial [20, 30, 31]. Accurate identification can be aided by morphological and immunoreactive differences, such as CD68 expression in osteoclast-like giant cells or beta-HCG in trophoblastic giant cells. It is also important to consider the possibility of metastasis from another organ or melanoma, depending on the clinical context [2, 26].

Due to of the rarity of GCUC, the lack of molecular characteristics of the disease is a challenge [32–34]. However, a potential response to targeted therapies has been suggested by preliminary data from lung cancer patients. Surgery is recommended for early-stage patients. In more advanced cases, MEK inhibitors, CDK4/6 inhibitors, and TP53 inhibitors are used [16]. A similar approach may also be possible for GCUC, although this is subject to the availability of further data.

A literature search of the PubMed database identified 29 previously reported cases [2, 4, 7, 8, 35–39]. Approximately 60% of patients succumbed or remained alive with the disease within 2 years regardless of therapy. Therefore, to understand optimal treatment strategies and to address the differential diagnostic challenges associated with this aggressive form of bladder cancer, original data from larger series are essential.

In this context, we present the clinicopathological features and oncological outcomes of the largest prospectively identified cohort of 23 cases of GCUC (21 patients). In addition, a comparison with cases of conventional UC matched by stage category is provided to delineate the differences between the two types of bladder cancer.

## Material and methods

A prospectively maintained database was used to conduct an observational study. A total of 23 cases from 21 patients diagnosed with GCUC were retrieved from the pathology archive of our institution. Available clinical information was obtained from the patient’s medical records. An average of 15 (range, 1–29) H&E-stained slides of routinely formalin-fixed, paraffin-embedded material from each case were systematically re-evaluated by a specialized genitourinary pathologist (ALB), who identified GCUC cases for inclusion in the database. GCUC was typically characterized by giant bizarre cells with pleomorphic nuclei identified on H&E-stained glass slides.

The histological evaluation of the samples also included the assessment of the associated conventional UC and its pathological grade. The percentage of GCUC that was present in each case was recorded. Other pathological features that were recorded included the presence of divergent differentiation or other variants (subtypes), lymphovascular invasion, stromal reaction, tumor necrosis, and the presence of perineural invasion.

The latest revision of the World Health Organization’s classification of the urinary system and male genital organs was used for the pathological classification of the tumors [1]. The cases spanned a period of 8 years. The first case was diagnosed in 2014 and the last in 2022. The follow-up period ranged 1–47 months (mean, 15±3 months; median, 13 months). The demographic characteristics of the patients as well as the stage category (pTNM or cTNM; AJCC/TNM 8th edition [40]) at diagnosis of bladder cancer and/or GCUC, the treatment(s) received before or after the diagnosis of GCUC, and the clinical outcome were also assessed.

For survival analysis (overall survival and cancer-specific survival), our case series of 21 patients (23 cases) was compared with a cohort of 119 patients with conventional UC who were assembled with randomly selected cases diagnosed in our institution over the same period in which there was 5 years minimum follow-up.

Immunohistochemical studies were performed on selected representative 4-μm paraffin sections (at least one section per case) to address specific differential diagnostic considerations and included GATA3 (Cell Marque, clone L50-823, prediluted), uroplakin II (clone BC-21), S100P (clone D28-E), PSA (clone 35H9, prediluted), NKX3.1 (clone EP356, prediluted), INI1 (clone MRQ-27, prediluted), CK20 (clone Ks20.8, prediluted), CK14 (Cell Marque, clone LL002, 1:300 dilution), CK5/6 (clone D5/16B4, prediluted), CK7 (clone RN7, prediluted), beta-HCG (Leica, polyclonal, prediluted), PAX8 (GenomeMe, clone IHC008, prediluted), p40 (clone BC28, prediluted), p53 (clone DO-7, (Leica, clone 27G12, prediluted), Ki67 (clone K2, prediluted)), and PDL1 (test 22C3). Immunohistochemistry was performed using either the Ventana Benchmark or Leica Bond platforms according to standard protocols for a given antibody. All analyses included appropriate negative and positive controls. Antigen retrieval was performed according to standard protocols when necessary. Immunostaining was graded on a scale from 0 to 3^+^.

To identify all reported cases of GCUC, a PubMed database search (www.pubmed.gov) was performed. The search terms used were giant cell bladder cancer, pleomorphic giant cell bladder cancer, giant cell urothelial carcinoma, pleomorphic giant cell urothelial carcinoma, and giant cell carcinoma. The search is up to date as of 31 January 2023.

### Statistical analysis

Data are presented as (i) proportions and frequencies when categorical and (ii) mean ± standard deviation, median when continuous. The distribution of overall survival and cancer-specific survival was estimated using the Kaplan–Meier analysis and Cox multivariate analysis. Analyses were performed using IBM SPSS Statistics v.26 for Windows software (IBM Corp, Armonk, NY). Results were considered statistically significant if the *P*-value was less than 0.05.

## Results

Clinicopathological features of 23 cases of GCUC identified in 21 UC patients are shown in Table 1. Patients were predominantly male, representing 86% (18/21). The age range was 65–88 years (mean, 74±1.5 years; median, 70 years). A history of previous UC was present in 52% of patients (11/21). The time interval between the initial diagnosis of conventional UC and the identification of the GCUC subtype was 40±19 months (median, 23 months). At the time of GCUC diagnosis, most patients were classified as AJCC stage I (30%; 7/23) or stage II (26%; 6/23). Stages IIIA and IIIB each accounted for 13% (3/23) of cases. In 9% (2/23) of patients, stages IVA and IVB were diagnosed. The most common diagnostic procedure was transurethral resection of bladder tumor in 83% (19/23) of cases. Radical cystoprostatectomy was performed in one case (4%). Lung, liver, and nodal metastases were each diagnosed in one case (4%). Associated conventional UC was identified in all GCUC cases. All cases were considered high grade. Carcinoma in situ was present in 22% of cases (5/23), and aberrant differentiation was noted in 48% of cases (11/23). The proportion of GCUC ranged from 10 to 30% (mean, 20±2%; median, 20%). Follow-up data were available for all patients, with 48% (10/21) succumbing to the disease at a median of 17±5 months (median 10 months); 24% (5/21) were alive with the disease; and 29% (6/21) had no evidence of disease at a mean follow-up of 9 months (range, 1–31 months).

**Table 1:** Demographic characteristics and clinicopathologic features of giant cell carcinoma subtype and associated conventional urothelial carcinoma of the bladder

Patient	Case^a^	Age (y)/gender	History of UC^b^; time to GCUC (mo)	GCUC %; sample type	Associated conventional UC	Pathologic features	IHQ UC component	IHQ GCUC component	TNM/AJCC at GCUC diagnosis	GCUC treatment(s) after diagnosis	Outcome (mo)
1	1	84/M	HGUC; 61	30%; liver metastasis biopsy	HGUC (G3)	LVI; SR; necrosis (20%)	GATA3+; S100P+; PSA-; NKx3.1-	GATA3- 100%; S100P 60%	M1b (IVB)	-	DOD (1)
2	2	82/M	HGUC; 23	10%; TURBT	HGUC (G3)	Squamous dif. (20%) LVI; necrosis (30%); peri- and intraneural invasion	-	-	cT4acN2M0 (IIIB)	CT + RT	DOD (4)
3	3	68/M	No; N/A	30%; TURBT	HGUC (G3)	Sarcomatoid (20%); Necrosis (10%)	-	-	pT2cN0M0 (II)	CyP	DOD (34)
	4		N/A	30%; nodal metastasis biopsy	HGUC (G3)	Necrosis (10%)	GATA3+; S100P+; INI1+; CK20+; CK 5/6-	S100P 60%; INI+; GATA3 100%; CK 5/6-; CK20 80%	M1a (IVA)	CT	
4	5	70/M	No; N/A	30%; TURBT	HGUC (G3)	Necrosis (20%)	CK 5/6-; CK20+; BHCG-	CK 5/6-; CK20 100%; BHCG-	cT1NxMx (I)	TURBT	NED (31)
	6		N/A	20%; TURBT	HGUC (G3)	Intestinal metaplasia; LVI, focal SR, necrosis (30%)	GATA3+CK20+; CK 5/6-; p53+ E-cadherin+	GATA3+; CK20+; CK 5/6-; p53+; E-cadherin+	pT2cN0M0 (II)	NAC + CyP	
5	7	81/M	HGUC; 185	30%; lung metastasis biopsy	HGUC (G3)	LVI, SR, necrosis (30%)	Synaptophysin-; S100P+; INI1+	S100P (100%)	cT4aN3M1b (IVB)	ICI	DOD (4)
6	8	70/M	No; N/A	30%; TURBT	HGUC (G3)	Squamous dif. (60%); SR; necrosis (10%)	PSA-; NKX3-; ki67+ 30%; p53+ 30%; S100P+; CK5/6+; CK20-	CK 5/6 100%; S100P 80%	pT4acN2M0 (IIIB)	ICI + RT; CT	DOD (21)
7	9	88/M	HGUC; N/A	10%; TURBT	HGUC (G3)	Small cell variant (60%); necrosis (50%)	Synaptophysin+ CK7+; GATA3+ focal; S100P+ focal; p63-	Synaptophysin neg; S100P 10%; GATA3 60%; p63 neg	cT3bN0M0 (IIIA)	CT + RT; TURBT; CT	DOD (38)
8	10	80/F	No; N/A	20%; TURBT	HGUC (G3)	LVI; necrosis (90%)	-	-	cT1NxMx (I)	-	DOD (13)
9	11	68/M	HGUC; N/A	20%; CyP	HGUC (G3)	Squamous (20%); LVI; SR; necrosis (30%); peri- and intraneural invasion	-	-	pT4aN2Mx (IIIB)	-	DOD (4)
10	12	86/M	HGUC; 27	20%; TURBT	HGUC (G3)	Squamous (10%); SR necrosis (10%)	CK7+; E-cadherin-; INI1+; CK5/6-; CK20-; IDO-1-; PDL1 POS<10	-	cT3bN0M0 (IIIA)	RT	AWD (20)
11	13	81/M	HGUC; 1	20%; TURBT	HGUC (G3)	-	-	-	pT2N0Mx (II)	RT	DOD (7)
12	14	70/M	N/A	10%; TURBT	HGUC (G3)	SR	-	-	pT2NxMx (II)	-	NED (1)
13	15	67/M	No; N/A	10%; TURBT	HGUC (G3); CIS	“Nested” cell (30%)	-	-	cT1NxMx (I)	TURBT + BCG; CyP + ACT	DOD (47)
14	16	69/M	HGUC; 20	30%; TURBT	HGUC (G3)	LVI, SR, necrosis (20%)	CK7+;CK20+; GATA3+	CK20 + (100%) CK7 + (100%)	pT2cN0M0 (II)	RT; TURBT; CyP	AWD (28)
15	17	65/F	N/A	10%; TURBT	HGUC (G3)	-	GATA3+; CK14+; p63+; p40+; CK 5/6+; Pax8-	-	pT2cNxMx (II)	-	NED (1)
16	18	74/M	HGUC; 9	20%; TURBT	HGUC (G3); CIS	-	-	-	cT3bN0M0 (IIIA)	-	AWD (5)
17	19	70/F	No; N/A	10%; TURBT	HGUC (G3)	Squamous (10%);	-	-	cT1NxMx (I)	TURBT + BCG; TURBT	AWD (20)
18	20	71/M	HGUC; 30	20%; TURBT	HGUC (G3); CIS	-	-	-	cT1NxMx (I)	BCG	NED (19)
19	21	71/M	No; N/A	20%; TURBT	HGUC (G3)	Squamous (10%); LVI; necrosis (10%)	CK5/6+; GATA3+; p63+; PDL1+ (20)	-	cT3bcN2M1a (IVA)	CT; ICI	AWD (22)
20	22	69/M	No; N/A	10%; TURBT	HGUC (G3); CIS	Glandular 20%; micropapillary 50%	P53+ 40%; Ki67+ 40%; GATA3+ 90%; Uroplakin+ 30%	Uroplakin+ 60%; P53; Ki67; and GATA3 100%	cT1N0M0 (I)	CyP	NED (1)
21	23	66/M	HGUC; 7	10%; TURBT	HGUC (G3); CIS	Glandular 50%; Micropapillary 10%	-		cT1NxMx (I)	-	NED (1)

*UC*, urothelial carcinoma; *GCUC*, giant cell urothelial carcinoma; *IHQ*, immunohistochemistry; *M*, male; *F*, female; *HGUC*, high-grade urothelial carcinoma; *TURBT*, transurethral resection of bladder tumor; *CyP*, cystoprostatectomy; *CIS*, carcinoma in situ; *LVI*, lymphovascular invasion; *SR*, stromal reaction; *Dif.*, differentiation; *LUTS*, low urinary tract symptoms; *NAC*, neoadjuvant chemotherapy; *CT*, chemotherapy; *ACT*, adjuvant chemotherapy; *RT*, radiation therapy; *ICI*, immune checkpoint inhibitor; *BCG*, bacillus Calmette–Guérin; *DOD*, died of the disease; *DOC*, died of other causes; *AWD*, alive with disease; *NED*, no evidence of disease; *LF*, lost in follow-up; *Y*, years; *mo*, months. ^a^All cases presented with hematuria except for case 9 which presented with storage LUTS. ^b^All previous UC cases were bladder UC

**Fig. 1:**
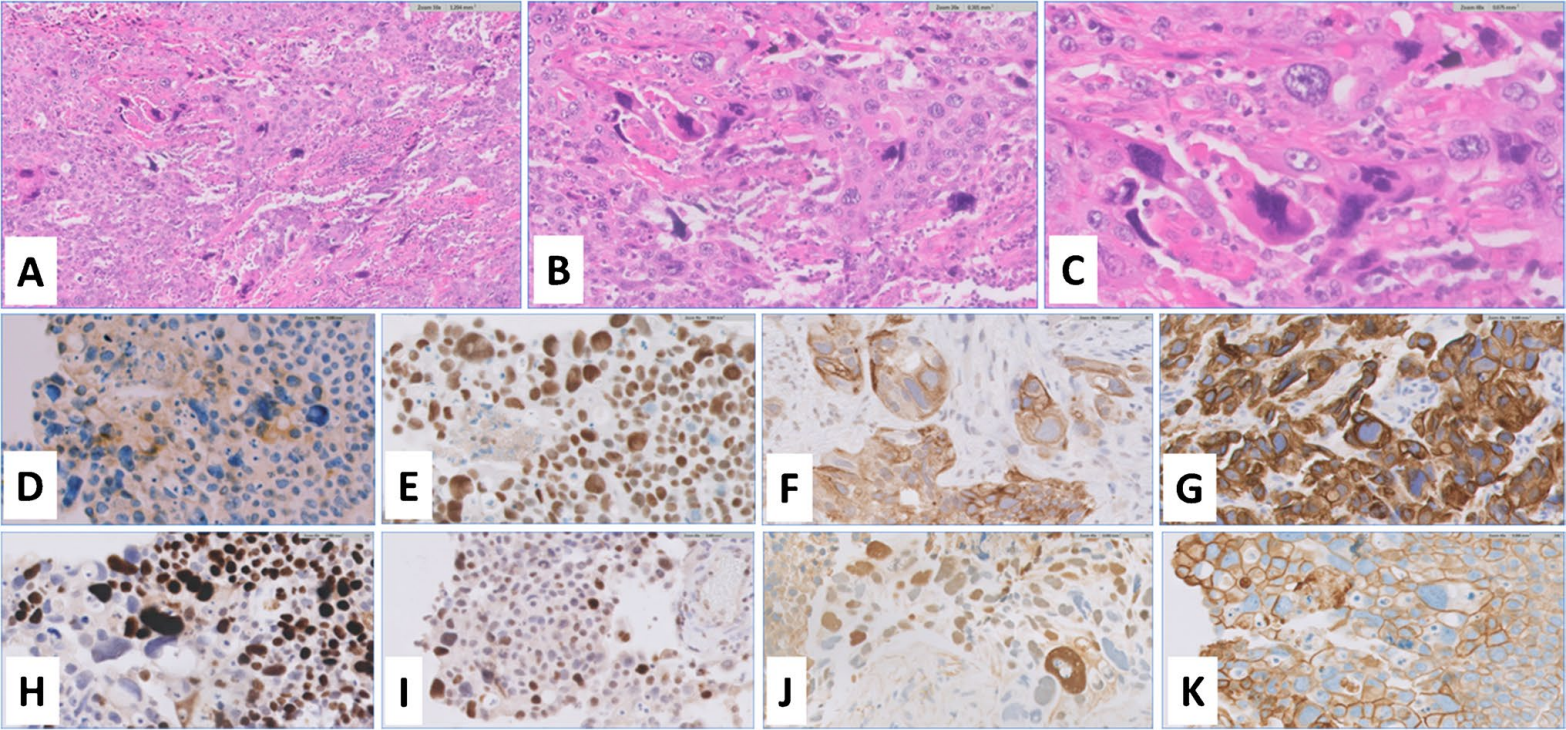
Representative features of giant cell carcinoma of the bladder with highly pleomorphic cells and largely variable hyperchromatic nuclei at low (**A**), intermediate (**B**), and high power (**C**) (**A**, **B**, **C** hematoxylin and eosin staining). Urothelial lineage, proliferation, p53, and cytokeratin immunohistochemical markers are readily expressed by cells in giant cell carcinoma uroplakin II (**D**), Gata3 (**E**), CK20 (**F**), CK7 (**G**), Ki67 (**H**), p53 (**I**), and S100P (**J**). PD-L1 expression with 22C3 antibody is also shown (**K**)

Immunohistochemical staining showed a similar profile for both GCUC and associated conventional UC (Fig. 1). It was characterized by a urothelial lineage with frequent positive expression of uroplakin II (focal), GATA3, CK20, CK7, and S100P. A high proliferation rate of Ki67 (range, 60–90%; mean, 71%) and an accumulation of p53 in the nucleus (mutant profile; range, 50–90%; mean, 64%) were observed. In the 22C3 assay, PD-L1 expression was variable in two cases and beta-HCG was negative. Other markers (INI1^+^; CK5/6^–^; E-cadherin^+^, synaptophysin^–^, p63^–^, PSA^–^, NKX3.1^–^, CK14^–^, PAX8^–^, p40^–^) used in selected cases gave results consistent with GCUC.

**Fig. 2:**
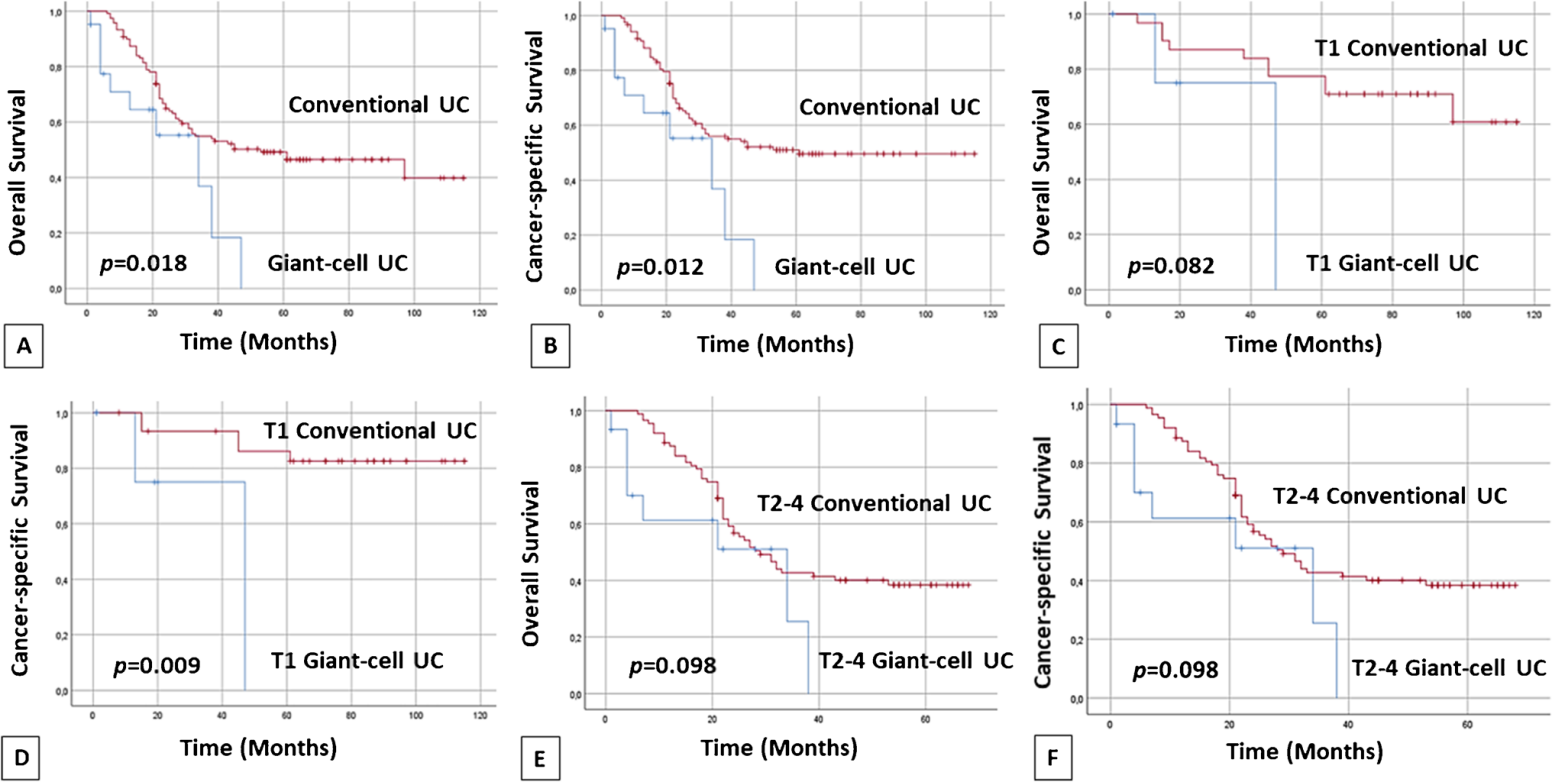
Kaplan–Meier plots showing overall and cancer-specific survival differences for giant cell carcinoma subtype vs. conventional urothelial carcinoma (**A**, **B**), stage T1 category for giant cell carcinoma subtype vs. conventional urothelial carcinoma (**C**, **D**), and AJCC stage T2-4 giant cell carcinoma subtype vs. conventional urothelial carcinoma in the current series of giant cell carcinoma subtype and conventional urothelial carcinoma (**E**, **F**)

The characteristics of the conventional UC series of 119 cases used in the current study for comparison purposes are summarized in Table 2. Table 3 shows the univariate analysis of survival using the log-rank and Kaplan–Meier plots. Significant overall and cancer-specific survival differences were observed when comparing GCUC with conventional UC (Fig. 2). This was particularly evident in the T1 stage category. Borderline significance was observed for overall survival and cancer-specific survival (both *P* = 0.098) in T2–4 cases. Table 4 indicates that GCUC and stage classification were both independent predictors of OS and CSS in multivariate analysis. The percentage of GCUC component or history of UC showed no significant association with survival in the current study. Table 5 shows the characteristics of previously reported cases of GCUC compared with our case series of 23 cases in 21 patients.

**Table 2:** Clinicopathologic features of giant cell carcinoma subtype as compared with conventional urothelial carcinoma in the current series

	GCUC (*n*=21)	Conventional UC (*n*=119)	*P*-value
Follow-up time (mean±SD, median)	15±3, 13	43±3, 38	< 0.001
T stage			0.161
T1, *n* (%)	6 (29)	31 (26)	
T2, *n* (%)	7 (33)	18 (15)	
T3a, *n* (%)	0	14 (12)	
T3b, *n* (%)	4 (19)	36 (30)	
T4a, *n* (%)	4 (19)	20 (17)	
Outcome			0.033
DOD, *n* (%)	10 (48)	56 (47)	
DOC, *n* (%)	0	6 (5)	
AWD, *n* (%)	5 (24)	6 (5)	
NED, *n* (%)	6 (29)	51 (43)	

*UC*, urothelial carcinoma; *GCUC*, giant cell urothelial carcinoma; *SD*, standard deviation; *DOD*, died of disease; *DOC*, died of other causes; *AWD*, alive with disease; *NED*, no evidence of disease

**Table 3:** Univariate survival analysis of selected clinicopathologic features in giant cell carcinoma subtype as compared to conventional urothelial carcinoma

	Total (*n*)	OS	Log-rank	*P*-value	CSS	Log-rank	*P*-value
Overall			5.628	0,018		6.384	0.012
GCUC, *n* (%)	21	11 (52)			11 (52)		
Conventional UC, *n* (%)	119	58 (49)			63 (53)		
T1			3.022	0.082		6.785	0.009
GCUC, *n* (%)	6	4 (67)			4 (67)		
Conventional UC, *n* (%)	31	21 (68)			26 (84)		
T2-4			2.742	0.098		2.742	0.098
GCUC, *n* (%)	15	7 (47)			7 (47)		
Conventional UC, *n* (%)	88	37 (42)			37 (42)		
GCUC %			1.735	0.420		1.735	0.420
10%, *n* (%)	8	5 (62.5)			5 (62.5)		
20%, *n* (%)	7	4 (57)			4 (57)		
30%, *n* (%)	6	2 (33)			2 (33)		
Previous History of UC			1.654	0.198		1.654	0.198
Yes, *n* (%)	11	5 (45.5)			5 (45.5)		
No, *n* (%)	8	4 (50)			4 (50)		

*UC*, urothelial carcinoma; *GCUC*, giant cell urothelial carcinoma; *OS*, overall survival; *CSS*, cancer-specific survival

**Table 4:** Multivariate survival analysis of selected clinicopathologic features in giant cell carcinoma subtype as compared to conventional urothelial carcinoma

	*P*-value	HR	95% CI	
OS				
Giant cell carcinoma	.021	2.222	1.126	4.384
Stage classification T1 vs. T2-4	.002	2.810	1.461	5.403
CSS				
Giant cell carcinoma	.015	2.328	1.176	4.608
Stage classification T1 vs. T2-4	.000	4.321	1.958	9.532

*OS*, overall-survival; *CSS*, cancer-specific survival; *HR*, hazard ratio; *CI*, confidence interval

**Table 5:** Salient clinicopathologic features of giant cell carcinoma subtype of the bladder obtained through literature search

Reference	No. of cases	Age range; (mean±SD; median)	Gender (%)	History of UC	GCUC %	Sample type	T stage	Staging AJCC	Outcome (mo) (range; mean±SD; median)
[2]	8	55–88; (67 ± 4; 62)	Male, 6 (75)	No	1 (100) 1 (50) 2 (30) 4 (20)	3 (TURBT) 3 (CyP) 2 (Cy)	2 (T4a) 4 (T3b) 2 (T3a)	8 (IIIA)	5 (DOD); (6–17; 11.2 ± 2; 10) 2 (AWD); (11–19; 15 ± 4; 15) 1 (NED); (74)
[36]	1	65	Male	No	1 (70)	1 (CyP)	1 (T3b)	1 (IVb)	1 (AWD); (4)
[35]	2	64–78; (71 ± 7; 71)	Female, 2 (100)	2 (N/A)	2 (N/A)	2 (TURBT)	2 (N/A)	2 (N/A)	2 (N/A)
[4]	13	53–93; (72±3.5; 73)	Male, 9 (69)	2 (bladder HGUC) 1 (ureteric HGUC) 10 (no)	3 (100) 1 (95) 3 (80) 3 (50) 3 (40)	11 (TURBT) 2 (CyP)	2 (T3b) 3 (T2) 8 (T1)	2 (IIIA) 3 (II) 8 (I)	5 (DOD); (2–12; 7 ± 2; 7) 4 (AWD); (15–34; 23.5 ± 4.5; 22.5) 1 (NED); (46) 3 (N/A)
[7]	1	82	Male	N/A	1 (100)	1 (TURBT)	1 (T1)	1 (I)	1 (NED); (12)
[37]	1	73	Male	No	1 (70)	1 (CyP)	1 (T3b)	1 (IIIB)	1 (NED); (48)
[8]	1	59	Male	No	N/A	1 (TURBT)	1 (T2)	1 (IVA)	1 (DOD); (15)
[38]	1	72	Male	Bladder HGUC	N/A	1 (CyP)	1 (T2)	1 (IIIA)	1 (NED); (58)
[39]	1	62	Female	No	1 (50)	1 (TURBT)	1 (T1)	1 (I)	1 (NED); (4)
Current study	23	65–88; (74 ± 1.5; 70)	Male, 18 (86)	11 (bladder HGUC) 8 (no) 2 (N/A)	7 (30) 8 (20) 8 (10)	19 (TURBT) 1 (CyP) 1 (liver met) 1 (lung met) 1 (nodal met)	4 (T4a) 4 (T3b) 7 (T2) 7 (T1) 1 (N/A)	2 (IVB) 2 (IVA) 3 (IIIB) 3 (IIIA) 6 (II) 7 (I)	10 (DOD); (1–47; 17 ± 5; 10) 5 (AWD); (5–28; 19 ± 4; 20) 6 (NED); (1–31; 9 ± 5; 1)
Summary of reported cases	52	53–93; (72 ± 1; 70)	Male, 38 (76)	30 (no) 14 (bladder HGUC) 1 (ureteric HGUC) 5 (N/A)	5 (100) 1 (95) 3 (80) 2 (70) 5 (50) 3 (40) 9 (30) 12 (20) 8 (10) 4 (N/A)	38 (TURBT) 9 (CyP) 2 (Cy) 1 (liver met) 1 (lung met) 1 (nodal met)	6 (T4a) 12 (T3b) 2 (T3a) 12 (T2) 17 (T1) 3 (N/A)	3 (IVB) 3 (IVA) 4 (IIIB) 14 (IIIA) 9 (II) 17 (I) 2 (N/A)	21 (DOD); (1–47; 13 ± 3; 10) 12 (AWD); (4–34; 19 ± 3; 19.5) 12 (NED); (1–74; 22 ± 7; 15.50) 5 (N/A)

*UC*, urothelial carcinoma; *GCUC*, giant cell urothelial carcinoma; *HGUC*, high-grade urothelial carcinoma; *TURBT*, transurethral resection of bladder tumor; *CyP*, cystoprostatectomy; *Cy*, cystectomy; *met*, metastasis; *DOD*, died of the disease; *DOC*, died of other causes; *AWD*, alive with disease; *NED*, no evidence of disease; *LF*, lost in follow-up; *mo*, months

## Discussion

Bladder cancer exhibit significant morphological heterogeneity and divergent differentiation [41]. This has led to the recognition of specific subtypes with unique histological appearances and diagnostic or prognostic implications [41]. Classic and recent reviews of unusual bladder cancer variants, including the current WHO classification of invasive urothelial tumors, have recognized the giant cell subtype of UC as a rare and aggressive variant characterized by the presence of highly pleomorphic and bizarre tumor giant cells, like those seen in giant cell carcinoma of the lung [1, 2, 4,, 7, 8, 35-39, 41]. However, when faced with the diagnosis of GCUC in routine pathology practice, we recognize that the limited data available, mostly derived from a few case reports and small case series, have led to a poor understanding of this disease and, more importantly, to variable clinical management and diagnostic uncertainty among pathologists [1, 2, 4, 7, 8, 35–39, 41].

Our series of 23 cases of GCUC represents the most extensive to date. It provides a detailed description of the architectural patterns, cellular features, immunohistochemical markers, clinical characteristics, and prognostic relevance of this rare form of UC. In our series, 70% of patients had a poor outcome, with 50% dying within 1 year of diagnosis, which is consistent with previously reported data. A review of 52 reported cases (Table 5) shows that 74% of reported patients died from or were alive with active disease [1, 2, 4]. The confirmed aggressiveness associated with GCUC supports the need for increased attention to improve our knowledge of this type of neoplasm, particularly regarding the molecular profile as a potential avenue for novel targeted therapies. At present, molecular data on this subtype of UC is limited. However, in line with clinical needs, Xi et al. [16] have demonstrated the potential benefits of targeted therapy for giant cell carcinoma of the lung based on molecular profiling, suggesting a possible survival benefit from MEK inhibitors, CDK4/6 inhibitors, and TP53 inhibitors. Although this study is a limited series, it opens the door to the study of GCUC and may contribute its inclusion in clinical trials of giant cell carcinomas in other organs, such as the lung. In addition, this clinical approach highlights the importance of accurate tumor classification, given the poor prognosis associated with GCUC. It also highlights the potential for novel therapies to treat affected patients [6, 32, 42–45]. An important finding in our study is the positive expression of PD-L1 in the two cases evaluated. They had tumor proportion scores of 10 and 20, respectively. Fortunately, after receiving the combination of pembrolizumab and radiotherapy, these two patients remained alive with the disease for 20 and 22 months, respectively.

Like other reported studies, 35% of patients in our series had advanced disease at presentation (stage III or IV), with distant metastases in 12% [2, 4, 8, 35, 36, 38, 39]. Observed differences in overall and cancer-specific survival when comparing GCUC with conventional UC indicate a trend to lower survival. This is an original finding that has not been reported previously, but the potential clinical impact of the survival analysis should not be overestimated due to the limited number of cases in the series.

The pathologist plays a crucial role in the diagnosis of GCUC. It is advisable to follow the WHO classification recommendations for the diagnosis of this entity [1]. Of potential importance is the distinction of GCUC from poorly differentiated subtypes, such as osteoclast-rich undifferentiated carcinoma and large cell undifferentiated carcinoma [20, 30]. Morphological and immunoreactive differences can aid in accurate identification. For example, CD68 expression is present in osteoclast-like giant cells and neoplastic giant cells are absent in large cell undifferentiated carcinoma. It is also important to distinguish GCUC from trophoblastic UC, which has trophoblastic giant cells as a landmark [31, 46]. The latter is less aggressive than GCUC, and trophoblastic giant cells typically express beta-HCG and other markers including GATA 3 by immunohistochemistry, which helps to make this distinction. It is important to consider the clinical context since metastases from other organs, particularly the lung or melanoma, may mimic GCUC. It should be noted that giant cell carcinoma arising in the prostate may spread to the bladder, particularly in patients on long-term treatment for prostate cancer, where the giant cell phenotype is not uncommon [12, 17]. This differential diagnosis is crucial, as the treatment approaches for advanced UC and advanced prostate cancer are quite different. In addition, accurate diagnosis may be facilitated by immunohistochemical panels that include markers for melanoma, lymphoid, trophoblastic, prostate, and urothelial lineage. Several urothelial lineage markers are expressed in GCUC but not in prostate giant cell carcinoma, giant cell carcinoma of other organs, or melanoma.

In conclusion, our study highlights the presence of GCUC, underlines its urothelial origin, and provides further evidence of its poor prognosis. The diagnosis can be challenging, especially in limited biopsy specimens, as it may be confused with secondary neoplasms or pleomorphic sarcomas. Histological features, consideration of the clinical context, and appropriate immunohistochemistry are essential to differentiate GCUC from mimics.
